# The Effects of a Physical Activity Intervention on Adiposity, Physical Fitness and Motor Competence: A School-Based, Non-Randomized Controlled Trial

**DOI:** 10.3390/children11010137

**Published:** 2024-01-22

**Authors:** Andrés Godoy-Cumillaf, Paola Fuentes-Merino, Frano Giakoni-Ramírez, Daniel Duclos-Bastías, José Bruneau-Chávez, Eugenio Merellano-Navarro

**Affiliations:** 1 Grupo de Investigación en Educación Física, Salud y Calidad de Vida (EFISAL), Facultad de Educación, Universidad Autónoma de Chile, Temuco 4780000, Chile; andres.godoy@uautonoma.cl (A.G.-C.); paola.fuentes@uautonoma.cl (P.F.-M.); 2 Faculty of Education and Social Sciences, Universidad Andres Bello, Las Condes, Santiago 7550000, Chile; frano.giakoni@unab.cl; 3 Escuela de Educación Física, Pontificia Universidad Católica de Valparaíso, Valparaíso 2340000, Chile; daniel.duclos@pucv.cl; 4 IGOID Research Group, Physical Activity and Sport Science Department, University of Castilla-La Mancha, 45071 Toledo, Spain; 5 Departamento de Educación Física, Deportes y Recreación, Universidad de la Frontera, Temuco 4811230, Chile; jose.bruneau@ufrontera.cl; 6 Department of Physical Activity Sciences, Faculty of Education Sciences, Universidad Católica del Maule, Talca 3530000, Chile

**Keywords:** cardiorespiratory fitness, strength, speed/agility, preschool, gross motor competence

## Abstract

Evidence suggests that early physical activity interventions are a means of preventing childhood obesity and are more effective when delivered in a school setting and based on the ecological model. Therefore, the present study aims to determine the effect of a multicomponent intervention based on the ecological model on adiposity, physical fitness and motor competence in children aged 4 to 5 years. Methods: This study is a non-randomized controlled trial involving 173 children from Chile. The intervention was based on an ecological model and consisted of a physical activity program with three simultaneous parts, affecting intra- and interpersonal dimensions. The adiposity index, body mass index and waist circumference were measured. For physical fitness, muscle strength in the lower part, speed/agility and cardiorespiratory fitness were measured. Motor competence was assessed using catching, aiming and dynamic and static balance tests. Results: After the intervention, there was no reduction in adiposity indices; in the intervention group, body mass index increased significantly with a high effect size. The intervention group showed significant differences in physical fitness in the components of muscle strength in the lower part (*p* = 0.000) and speed/agility (*p* = 0.002). For motor competence, the intervention group showed significant improvements in most components. Conclusions: The multicomponent intervention did not reduce adiposity indices; however, it caused significant improvements in the physical fitness and motor competence components, so it seems prudent to continue implementing it, given the benefits that adequate levels of motor competence and physical fitness bring to children’s health, both in the short and long term.

## 1. Introduction

Obesity has now become a global public health problem [1], affecting mainly low- and middle-income countries [2,3]. There is currently a debate about what approach should be taken to manage and prevent childhood obesity, as public policies have shown little effectiveness in reducing obesity rates worldwide over the last three decades [4]. Some researchers point to the importance of physical activity in controlling and preventing weight gain [5], while others point to increased investment in tackling poor nutrition in the population [6]. However, the scientific community is calling for the development of promotion and prevention strategies through physical activity at school age.

In Chile, the data are not encouraging, with 56.5% of 4-year-olds, 59.6% of 5-year-olds and 59.3% of 6-year-olds being overweight/obese [7], percentages that increase as children enter adolescence and adulthood [8,9]. Increased sedentary behavior has been identified as one of the factors contributing to increasing levels of obesity [6,10]. To address this problem, it is necessary to focus on factors that help reduce sedentary behavior [5] and improve physical fitness and motor skills during childhood [11].

Physical fitness (PF) is a measure that integrates most of the functions of the human body and is associated with the ability to participate in various forms of physical activity [12]. Adequate levels of PF are considered important for the health of children and adolescents, mainly because they reduce the prevalence of cardiovascular disease, mental illness and obesity [13,14]. For preschool children, although the information is not as robust as for children and adolescents, there is evidence that lower levels of adiposity are associated with good levels of PF [15,16]. On the other hand, there is evidence that FC in this age group is associated with muscle and bone development [17], mental health [18] and cognitive performance [19].

On the other hand, motor competence (MC) is the degree to which an individual is able to perform a variety of motor tasks with good body control and adequate coordination of movements and the mechanisms underlying performance [20]. Evidence on MC suggests that its adequate development from preschool age contributes to adequate cardiorespiratory endurance and muscular fitness, better cognitive development and academic performance and a healthy weight [11,21,22].

Despite the health benefits of having adequate levels of PF and MC, chilean children have low levels of both [23,24], which, combined with their non-compliance with physical activity recommendations [25,26], places them in a worrying situation due to the reciprocal relationship between high levels of adiposity and physical activity, PF and MC [11]. In this sense, physical activity interventions applied from an early age are a means of preventing childhood obesity and are more effective when implemented in a school setting [27,28,29]. Therefore, designing and implementing strategies from an early age is key to achieving long-term outcomes in PF and MC [30]. On the other hand, evidence suggests that to be more effective, interventions need to be designed from a socio-ecological perspective of the problem of physical inactivity, which requires a view that includes the school and family environment [31,32,33].

Based on the ecological model of physical activity, numerous international studies have analyzed the influence of school environment characteristics on physical activity levels [27,33,34]. As a result, the possibilities for analyzing this model are numerous, allowing the school to be considered as a specific unit of analysis that interacts with the school environment, the educational community and, in particular, the family [30,35]. From this perspective, the research question was born, asking what the results of a physical activity intervention with articulation between school and family on adiposity indices, physical fitness and motor competence in boys and girls will be.

The aim of this research was to determine the effect of a multicomponent intervention based on the ecological model on adiposity, physical fitness and motor competence in children aged 4 to 5 years. It is hypothesized that following the intervention, adiposity indices will decrease and physical fitness and motor competence scores will improve. It is hoped that the results of this study will contribute to the development of programs that promote greater physical activity from an early age.

## 2. Materials and Methods

### 2.1. Study Design

This study is a non-randomized controlled trial involving 173 children aged 4 and 5 from an educational institution in a city in southern Chile. The application of the assessment instruments and the study intervention were carried out between March and September 2019.

### 2.2. Participants

The sample was of non-probability convenience, with the inclusion criteria (a) being between 4 and 5 years of age, (b) being in the pre-kindergarten and kindergarten levels of preschool education, (c) having parental consent to participate in the intervention and (d) having the consent of the child when asked to participate. The exclusion criteria were (a) having musculoskeletal complications or mental and/or chronic illnesses that prevented participation in the program, (b) not participating in the pre- or post-intervention assessments and (c) not reaching 80% participation in the program. The Universidad Autónoma de Chile, through its Scientific Ethics Committee, approved the study protocol (N°11–19) and registered it on ClinicalTrial.gov, access date 9 March 2019 (NCT04194580). A total of 116 children formed the intervention group, and 57 formed the control group.

### 2.3. Intervention

The design of the physical activity program was based on a study with similar aims to the present study [36]. The intervention, based on the ecological model, lasted 5 months (April to August 2019) and consisted of a physical activity program composed of three simultaneous parts, focusing on intrapersonal and interpersonal dimensions. The first part (intrapersonal) consisted of different games played outside school hours (traditional, pre-sports, dances), performed at moderate to vigorous intensity [37], lasting 45 min per session, three times a week. The second part (interpersonal) consisted of educational activities to promote physical activity among parents and teachers. Parents received monthly 15 min educational talks on the following topics: what physical activity is and the benefits it brings throughout life; the benefits of physical activity in preschool children; the recommended amount of physical activity and current levels for the Chilean population; and the health risks of not doing the recommended amount of physical activity. In addition, the topics were reinforced through short videos sent to parents once a week via social networks. Teachers used the same themes with the children as with the parents, but through the creation of products (handicrafts such as drawing and painting) and through storytelling. The frequency of these activities was three times a week.

The third part consisted of physical activity at school, outside of physical education class or the intervention program, which took place when the children arrived and left school, during breaks and when moving from one room to another, using balance and coordination circuits on the floor. The frequency of these activities was daily. The control group did not receive the after-school physical activity program but received the second and third parts.

The out-of-school physical activity program was delivered by a physical activity graduate who was trained to ensure the standardization of the sessions. Both the control and intervention groups received their regular physical education classes once a week for 45 min.

### 2.4. Study Variables

The study variables were assessed before and after the implementation of the program (March and September 2019). All physical assessments were carried out by the researcher in charge, in collaboration with a group of physical education teachers external to the educational center who were trained to guarantee standardization, and with the support of the early childhood educator corresponding to each level evaluated.

#### 2.4.1. Adiposity Indices

Body mass index: Height was measured with a stadiometer and weight with a digital scale (Seca 222 and Tanita MC 780U, respectively). Both were measured in duplicate, and the mean of each was used to calculate the body mass index by dividing weight by height squared (kg/m^2^).

Waist circumference: The last rib and the top of the iliac crest were located, and the midpoint between the two measurements was taken. Using a tape measure (Rosscraft), the value was determined at the end of a normal expiration. It was assessed three times, and the average was calculated.

#### 2.4.2. Physical Fitness

It was determined by tests that are part of the PREFIT battery [38]. This is an instrument that has been shown to be easy to use, feasible and highly reliable in preschool children [39] and has been used in a population aged 3–5 years in Chile [29,40]. Cardiorespiratory fitness, muscular strength in the lower part and speed/agility were assessed using the 20 m shuttle run, long jump and 4 × 10 sprint tests. In order to avoid potential errors in data collection, in addition to strict standardization, recommendations proposed by the authors were followed, such as: the most appropriate sequence of tests; that children wear comfortable sports clothing and appropriate shoes; and, given the age of the participants, that they receive constant messages of encouragement and motivation to achieve maximum performance [38].

#### 2.4.3. Motor Competence

It was assessed using the Movement Assessment Battery for Children, second edition (MABC-2) [41], which has been shown to be an instrument with adequate psychometric reliability properties (Cronbach’s α > 0.60; κ = 1; ICC = 0.85–0.99) [42], using the validated version for Spanish [42]. The gross motor competence tasks of catching and aiming (2 tests) and static and dynamic balance (3 tests, one-leg balance, tiptoe walking, floor mat) were used to assess gross motor competence.

### 2.5. Statistical Analysis

First, the Kolmogorov–Smirnov test was used to check the normality of the data, after which parametric tests were used for the effect analyses. Before the start of the intervention and as a pre-test, comparative analyses between the groups were carried out using Student’s *t*-test for independent samples to check the homogeneity of the groups in the variables related to this study. At the end of this study and as a post-test, differences between subjects were analyzed using Student’s *t*-test for paired samples, and differences between groups were again analyzed using Student’s *t*-test for independent samples. Statistical significance was set at *p* < 0.05 for all tests. For variables where significant differences were found, the Cohen’s d test was used to determine the effect size, with the following scale of interpretation: ≥0.2 small; ≥0.5 moderate; and ≥0.8 large [43]. The data were analyzed using the SPSS statistical package, version 29.

## 3. Results

The descriptive characteristics of the sample at the beginning of this study and the values obtained at the end of the program are presented in Table 1. In terms of adiposity indices, the groups were homogeneous at the beginning of the activity. After application of the program, BMI increased in the intervention group (from 17.82 to 19.04), with differences that were statistically significant (*p* = 0.000) with a high effect size (1.20). This increase also occurred in the control group (from 17.33 to 18.27) with significant values (*p* = 0.005), although with a smaller effect size (0.39). For waist circumference, increases occurred in the control group (from 55.71 to 56.71) and in the intervention group (from 57.43 to 57.62), but only in the control group were the values significant (*p* = 0.021), with a small effect size (0.31).

In terms of physical fitness, the groups were homogeneous at the start of the activity. At the end of the program, the intervention group showed an increase in muscle strength in the lower part (from 79.56 to 86.83), which was significant (*p* = 0.000), and a decrease in speed/agility (from 17.01 to 16.63), which was also significant (*p* = 0.002). The control group also showed improvements in muscle strength in the lower part (from 80.51 to 87.84) and speed/agility (from 17.61 to 16.88), both with significant differences (*p* = 0.001 and *p* = 0.001, respectively). In all cases, the effect size was small.

With regard to gross motor competence, the groups were not homogeneous at baseline. After the program, the intervention group showed significant improvements in the catching (*p* = 0.001), aiming (*p* = 0.000), right balance (*p* = 0.000), left balance (*p* = 0.000) and floor mat (*p* = 0.000) tests. The control group also showed significant improvements in the catching (*p* = 0.003), right balance (*p* = 0.000) and left balance (*p* = 0.002) tests. The effect size was small in all cases.

## 4. Discussion

The aim of the present study was to determine the effect of a multicomponent intervention based on the ecological model on adiposity, physical fitness and motor competence in children aged 4 to 5 years. The main findings were that the intervention failed to reduce adiposity indices; however, it led to significant improvements in the components of PF and gross motor competence. The control group also showed significant differences in some of the variables studied.

The results of the intervention on adiposity were not as expected, because boys and girls gained 1.2 kg/m^2^ after the intervention. In relation to this finding, studies that have examined the effectiveness of physical activity interventions on adiposity rates in preschool children have reported mixed results. Specifically using BMI as an indicator, Waters et al. [44] reported reductions in children under 5 years of age but concluded that obesity prevention programs are particularly beneficial for children between 6 and 12 years of age. A preschool intervention program in Switzerland with immigrant children reported reductions in BMI and body fat percentage [45]. On the other hand, a two-year program in preschool children reported no reductions [46], and a similar situation was reported by Martínez-Vizcaíno et al. [30]. Our study also found no reductions in adiposity-related indices, so while no one is questioning its implementation, we believe that so far it confirms that the evidence for the effectiveness of physical activity interventions on adiposity remains moderately strong [29,47]. Furthermore, although there is evidence that BMI is a useful and practical measure for classifying adiposity [48], in children and adolescents, it may be a poor measure for accurately reflecting changes in adiposity over time [49,50].

On the other hand, waist circumference increased significantly in the control group (0.021 cm). It should be noted that this indicator is considered to be a more valid measure of central obesity than BMI [51,52] and is considered to be a predictor of increased blood pressure in preschool and school-aged children [53,54,55,56]. For this reason, we believe that the group that did not participate in the after-school physical activity intervention increased their waist circumference values due to increased abdominal fat, whereas the intervention group did not increase their waist circumference values due to the physical activity effect. However, the use of other techniques to measure body fat is needed to determine the changes more accurately.

In terms of physical fitness, the intervention group achieved significant improvements in muscle strength in the lower part and the speed/agility component. These results are important, as it has been shown that in children, muscle strength and the risk of cardiometabolic diseases are inversely related [57,58], and that better values bring benefits to the organism, such as greater bone mineral density and, at a later age, the accumulation of bone mass [59,60]. This situation is particularly relevant in the sample studied, since low levels of muscle strength have been reported in the Chilean child population [24], so a physical activity program could help to counteract this.

For the cardiorespiratory fitness component, the intervention group showed improvements, but these were not significant. The same situation was reported in a study [30] using the exercise program that was the basis of the present study [36]. In that study, the researchers hypothesized that the lack of improvement in cardiorespiratory fitness was because a longer intervention time (more than a year) is usually needed to achieve effects on this component [61], and therefore the total duration was not long enough (8 months). We believe that the same argument can be used to explain the results of the present study, as our intervention lasted 5 months.

With regard to the results of gross motor competence, the intervention group obtained significant improvements in five of the six tests evaluated (with the exception of tiptoe steps, *p* = 0.175), a situation that is in line with the evidence showing that interventions aimed at improving motor competence are effective both in the short and long term (more or less than 6 months) [62]. In addition, Morgan et al. [63] point out that interventions may be more effective if they are delivered by physical education specialists, a situation that occurred in the present study and seems to be one of the reasons explaining the results found.

The improvements obtained in gross motor competence are important because current evidence suggests that Chilean children have low levels of motor competence [23,24], which requires the application of more programs such as this one. Although the application of a program such as the one analyzed in this study is important and motor tasks should be repeatedly taught, practiced and reinforced for their correct development [64], the application of this type of intervention should be maintained over time, as follow-up studies with a duration between 3 and 12 months after the intervention report that the effects are not long-lasting [65,66].

On the other hand, the results for the control group showed that some components of PF and gross motor competence improved significantly despite not participating in the physical activity intervention. Although this group did not participate in the out-of-school physical activity intervention, which consisted of 45 min of moderate-to-vigorous intensity play three times a week, they participated in the second and third parts of the intervention, which consisted of physical activity promotion activities by parents and teachers and in-school physical activity using paths or obstacles located in different parts of the school. This situation could have led to a greater knowledge of the benefits of physical activity among the children in the control group, since parents are relevant for their children in the transmission of behaviors to be followed [67], which could have led to a greater practice of physical activity, which could have benefited from the use of the routes and obstacles placed in the school. However, a qualitative study is needed to know the exact role of parents.

It is reasonable to assume that the intervention group would have better results than the control group, as they received a higher dose of physical activity, but it is at this stage of development that gradual improvements in MC occur through individual predispositions and motor experiences [68,69], which can be influenced or refined during development [70]. However, for further analysis, it is necessary to consider some environmental components that were not investigated in this review and that may have influenced improvements in PF and gross motor competence scores, such as intensity of physical activity outside of school, participation in sports [71], quality of physical education experiences at school [72], socioeconomic status [73] and gender inequality [74], among others.

An interesting point to discuss is the effectiveness of the interventions, but leaving aside the significant differences, when analyzing the data, the cardiorespiratory fitness components of the intervention group and the static balance in both groups improved, but not significantly. This situation would not be highlighted in most studies because a *p*-value < 0.05 is always expected, but it indicates that what was implemented caused improvements. We believe that these data should also be highlighted, as they give us an indication of the benefits of the program implemented and allow us to give feedback to the program to achieve improvements, for example, in our case, giving more emphasis to activities where cardiorespiratory fitness or static balance work predominate.

The results of the present investigation are useful for the population of the sample studied, since although it did not produce changes in variables related to adiposity, it produced changes in physical fitness and gross motor competence, so it seems prudent to continue implementing them, given the benefits that adequate values of gross motor competence and physical fitness provide for the health of children, both in the short and long term [11,13,14,15,16,17,18,19,21,22]. The results obtained are also useful for future studies to be carried out not only in Chile but also in Latin America, since due to the particular complexity of the region, where the rates of overweight and obesity have increased in the last decades [75,76,77], we believe that the implementation of program designs that consider the ecological model of intervention, from a local perspective, through the conformation of multidisciplinary teams and the objective evaluation of physical activity variables, together with the monitoring of physical fitness and motor competence indicators, could allow the treatment and prevention of diseases related to poor health habits.

The strengths of this study are the following: The use of a non-competitive play intervention adapted to the characteristics of the children and involving teachers and parents. Its implementation in the school environment allows the participation of all pupils, regardless of gender, ability or physical fitness. The use of rigorous and standardized assessment instruments, not self-report. Planning the intervention based on a program that has been used previously [30] with positive results means the activities of each session are planned and structured. The limitations of this study are the following: The use of a non-probability convenience sample does not allow generalization of the results, so randomized controlled trials are recommended to confirm the benefits. The duration of the intervention (5 months) may have influenced the lack of improvement in some of the components assessed. Not subjectively or objectively measuring the total physical activity carried out by the children to determine the influence of the intervention and the physical activity carried out outside of school hours. It does not determine the prevalence of overweight, obesity and sedentary behaviors since these factors influence the modification of the variables studied. Finally, the environmental, political and social factors of the school environment in which the school was located were not considered as measurement variables.

## 5. Conclusions

The multicomponent intervention did not reduce adiposity indices; however, it caused significant improvements in the components of physical fitness and motor competence, which, if maintained over time, would benefit the health of those evaluated. It is recommended that the implementation of these interventions in preschool children be continued, together with a follow-up that will allow us to have solid evidence of the benefits of the interventions on the variables evaluated in this study.

## Figures and Tables

**Table 1:** Pre- and post-intervention differences in intervention and control groups.

	Control Group N = 57				Intervention Group N = 116			
	Pre-Test	Post-Test	*p*	*d*	Pre-Test	Post-Test	*p*	*d*
Adiposity indices								
BMI (kg/m^2^)	17.33 (±2.09)	18.27 (±3.31)	**0.005**	0.39	17.82 (±2.31)	19.04 (±2.75)	**0.000**	1.20
Waist circumference (cm)	55.71 (±6.25)	56.71 (±6.51)	**0.021**	0.31	57.43 (±6.37)	57.62 (±8.20)	0.772	0.03
Physical fitness								
Muscle strength of the lower part (cm)	80.51 (±19.38)	87.84 (±19.28)	**0.001**	0.48	79.56 (±23.35)	86.83 (±21.68)	**0.000**	0.38
Speed/agility ^¥^ (s)	17.61 (±2.20)	16.88 (±2.40)	**0.001**	−0.47	17.01 (±1.90)	16.63 (±1.66)	**0.002**	−0.30
CRF (stage)	1.54 (±0.66)	1.52 (±0.69)	0.598	−0.07	1.77 (±0.93)	2.42 (±5.15)	0.184	0.12
Gross motor competence, catching and aiming								
Catching (number)	7.47 (±2.66)	8.47 (±2.07)	**0.003**	0.42	7.89 (±2.33)	8.68 (±2.40)	**0.001**	0.32
Aiming (number) Balance Static balance	3.91 (±2.34)	4.25 (±2.21)	0.303	0.14	4.29 (±1.92)	5.66 (±3.62)	**0.000**	0.35
Right balance (seg)	13.56 (±8.94)	20.89 (±9.11)	**0.000**	0.73	17.34 (±10.32)	21.40 (±10.39)	**0.000**	0.45
Left Balance (seg) Dynamic balance	15.45 (±9.97)	19.78 (±10.08)	**0.002**	0.43	17.29 (±10.60)	21.06 (±10.26)	**0.000**	0.39
Tiptoes (steps)	12.96 (±4.25)	13.98 (±3.04)	0.071	0.24	13.66 (±3.43)	14.14 (±2.58)	0.175	0.13
Floor mat (jumps)	4.86 (±0.39)	4.88 (±0.50)	0.709	0.05	4.67 (±0.71)	4.93 (±0.32)	**0.000**	0.37

CRF cardiorespiratory fitness. ^¥^ Less time (in seconds) indicates better speed/agility levels. The values in bold indicate a statistical significance of *p* < 0.05.

## Data Availability

The data presented in this study are available on request from the corresponding author. The data are not publicly available due to ethical standards.
